# Sex- specific interplay of combined lifestyle patterns and their association with depressive symptoms among Chinese adolescents: a school-based cross-sectional study

**DOI:** 10.3389/fpsyt.2026.1747059

**Published:** 2026-05-12

**Authors:** Yi Lin, Jin-Ying Huang, Zeng-Bao Hu, Richard Rankin, Stuart McDonald, Ke-Qin Ding, Qing-Hai Gong, Guo-Lin Bian

**Affiliations:** 1 Faculty of Humanities and Social Sciences, University of Nottingham Ningbo China, Ningbo, Zhejiang, China; 2 College of International Economics & Trade, Ningbo University of Finance & Economics, Ningbo, Zhejiang, China; 3 Department of Science and Engineering Education, University of Nottingham Ningbo China, Ningbo, Zhejiang, China; 4 The Department of School Health, Ningbo Municipal Center for Disease Control and Prevention, Ningbo, Zhejiang Province, China; 5 Ningbo Municipal Center for Disease Control and Prevention, Ningbo, Zhejiang Province, China

**Keywords:** adolescents, depressive symptoms, lifestyle behaviors, screen time, sleep duration, sugar-sweetened beverage

## Abstract

**Background:**

Unhealthy lifestyle behaviors are known as risk factors for development of depressive symptoms among children and adolescents. Evidence shows sedentary behaviors, sleep duration (SLD), and diet are associated with depressive symptoms. The combination of these lifestyle behaviors may work together to affect mental health. However, the potential role of sex in this association remains unclear. This study aimed to investigate sex-specific associations between combined lifestyle of sugar-sweetened beverage (SSB), screen-based sedentary time (SST) and SLD, and depressive symptoms in Chinese adolescents.

**Methods:**

A school-based study was conducted in Ningbo from 2022 to 2023. A multistage, stratified cluster sampling procedure was used to draw adolescents aged 11–19 years. Depressive symptoms were identified by the Centre for Epidemiological Studies Depression Scale. Multilevel logistic regression was used to examine associations between depressive symptoms and combined lifestyle patterns.

**Results:**

In total, 19,057 adolescents were included for the final data analysis. The prevalence of depressive symptoms was more prevalent in girls (boys vs. girls: 14.54% vs.18.27%; P <0.001). The combination of low SSB consumption, excessive SST and short SLD and the combination of high SSB consumption, excessive SST and short SLD were associated with higher odds of depressive symptoms, while the combination of high SSB consumption, appropriate SST and sufficient SLD was associated with lower risk of depressive symptoms. When stratified by sex, the combination of low SSB consumption, excessive SST and sufficient SLD, and the combination of high SSB consumption, excessive SST and sufficient SLD were associated with depressive symptoms in girls (AOR: 1.48, 95% CI: 0.97**–**2.25) and boys (AOR: 0.66, 95% CI: 0.49**–**0.89), respectively.

**Conclusion:**

The association between lifestyle behaviors and depressive symptoms differs by sex. Our findings suggest that sex-specific patterns should be considered when designing future school-based health strategies aimed at promoting healthy lifestyle behaviors and supporting adolescent mental well-being.

## 1 Introduction

Depression is a common mental disorder and has become a major public health concern across all age groups in adolescents globally (1, 2). It was hypothesized that depression is one of the main causes of disability and illness, and a leading contributor to the global burden in adolescents (1, 3). The worldwide prevalence of depression in adolescents has risen sharply (4). World Health Organization (WHO) reported that, globally, 14% adolescents aged 10–19 years suffered from depression and other mental disorders (2). The estimated prevalence of depressive symptoms, concluded from a meta-analysis of 51 observational studies, was about 19.9% in Chinese adolescents (5).

Adolescence is a transitional period of physical and psychological development from childhood to adulthood, as well as the development of healthy behaviors and changes in lifestyle behaviors (2, 6). A recent systematic review concluded that meeting lifestyle recommendations in children and adolescents can benefit mental health and reduce the risk of mental disorders (7). Given the rapid development of economies and technology for the past 5 decades in China, the traditional diets and lifestyle have been shifted towards “Western” diets accompanied by an increase in sedentary lifestyles, resulting in low-quality lifestyle behaviors associated with psychological status, mental disorder and chronic diseases (8). Based on the guidelines for Chinese children and adolescents’ health status and diseases prevention (9, 10), approximately 85.8% of students aged 6–17 years engaged in prolonged sedentary lifestyle, 36.0% of middle-school students spent more than 2 hours per day on electronic devices and 80% of adolescents aged 8–16 years had less than the recommended sleep duration (SLD) (11–13). Around 65.0% of Chinese middle school students were reported to consume sugar-sweetened beverage (SSB) more than once daily (14).

Adolescents’ lifestyle behaviors play an important role in prevention and management of mental disorders. Evidence shows that excessive screen-based sedentary time (SST), short SLD and unhealthy dietary patterns were associated with increased risk of depressive symptoms in adolescents separately (15–17). Previous studies indicated that insufficient sleep was associated with an increase in mental disorders in adolescents (15, 18). Currently, Chinese adolescents have exposure and access to electronic devices nationwide. Screen-based sedentary behavior can contribute to the development of mental health problems in childhood (19). Increased SST was reported to be associated with the development of emotional problems and mental disorders among adolescents (20).

Evidence determines the importance of the relationship between the quality of dietary patterns, and depressive symptoms and mental health in children and adolescents (16). Previous studies concluded that SSB consumption is a major risk factor for developing depressive symptoms among adolescents (14, 21). Results from a longitudinal study showed that more frequent SSB consumption may contribute to aggressive behaviors over time (22).

Most previous studies link one certain lifestyle factor, such as sedentary lifestyle, sleep or diets, with adolescents’ depressive symptoms (16, 20). While integrative frameworks such as 24-hour movement behavior guidelines (physical activity, sedentary time, and sleep) have been well studied comprehensively understand how these multiple lifestyle behaviors are jointly associated with adolescent health (23), less attention has been paid to other co-occurring lifestyle behaviors such as dietary patterns, SST, and sleep duration, which fall outside well-established framework. Moreover, it remains unclear whether other co-occurrence of lifestyle behaviors is associated with depressive symptoms in adolescents. Therefore, the objective of this study is to estimate sex difference in the prevalence of depressive symptoms and identify the combination of SSB, SST and SLD, to investigate sex-specific associations between those combined lifestyle patterns and depressive symptoms among Chinese adolescents.

## 2 Methods

### 2.1 Study design and study participants

This school-based study was conducted in Ningbo, Zhejiang Province, China by the Ningbo Center for Disease Control and Prevention (CDC). Two repeated surveys were performed from September to October in 2022 and 2023, covering socio-economic status, demographic, and physical and mental health status.

**Figure 1:**
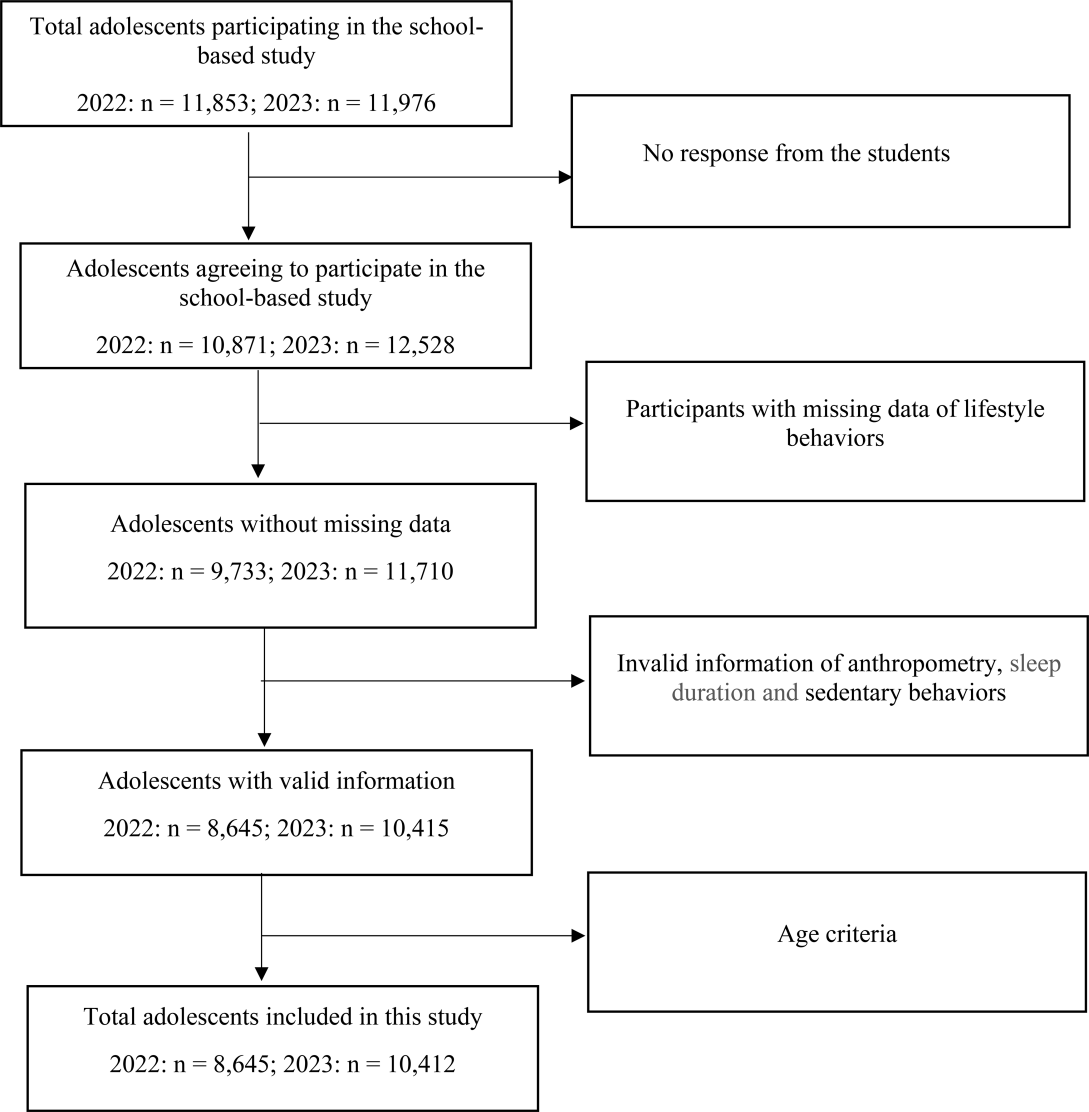
Flowchart of study population participating in this school-based study in 2022 and 2023.

A multistage, stratified cluster random sampling procedure was used to draw the target samples. Schools including at least two junior high schools, two senior high schools and one vocational high school were selected from each district, while at least two high schools and one senior high school were selected from each county at the first stage. Two classes were selected from each grade of the selected school at the second stage. A total of 23,829 high school students were contacted to participate in this study, of which 23,399 students participated in the repeated surveys, with a participation rate of 98.2% (**Figure 1**). A total of 1,956 students had missing data on lifestyles, with screen-based sedentary behavior exhibiting the highest proportion of missing values, followed by sleep duration. An additional 2,383 students had invalid information on anthropometry and lifestyles. Ultimately, 19,057 adolescents were included in this study. Details of study design were reported elsewhere (24).

Experienced researchers provided an introduction and instructions of the survey and emphasized the confidentiality of the questionnaire before starting the survey. Under the supervision of researchers, all students were asked to complete self-administered anonymous questionnaires independently in their classroom with no teachers present. All submitted information was double-checked for quality control by researchers.

The inclusion criteria for this study were: 1) students aged 11–19 years; 2) ability to understand the questions and complete the questionnaire independently; 3) the signed consent forms from a parent or legal guardian of the student. The exclusion criteria were: 1) students who refused to participate in the survey; 2) students who were sick or had injuries influencing the health examination; 3) students who did not attend the health examination.

This study was conducted in accordance with the Declaration of Helsinki, and approved by Ethics Committee of Ningbo CDC (No. 202201) and Ethics Committee of University of Nottingham Ningbo China. Written informed consent was obtained from students’ parents or legal guardians. All information related to students is confidential.

### 2.2 Measures and assessment

All the information was collected through a standard questionnaire used in this study were developed and approved by a panel of experts in accordance with the guidelines proposed by School Health Preparatory Office, Chinese CDC, and the study protocol followed the guideline by Zhejiang Provincial CDC (25).

Depressive symptoms for the past seven days were assessed using the Chinese version of Centre for Epidemiological Studies-Depression (CES-D), which are related to restless sleep, poor appetite, and mood that they experience. The Chinese version of CES-D consisting of 20 items, was validated previously and has been widely used on Chinese students with good reliability and validity (26, 27). Students were asked to report the frequency of each depressive symptom for the past week. Each item has 4 response options: 0-rarely or none of the time (<1 day), 1-some or little of the time (1–2 days), 2-moderately or much of the time (3–4 days), 3-most or almost all the time (5–7 days). The total score ranges from 0 to 60 with higher scores indicating greater depressive symptoms and a score of greater than or equal to 16 identifying individuals with depressive symptoms (28).

SSB was evaluated using the question ‘How often did you drink soda and other sugar-sweetened drinks including Coca Cola, Iced Black Tea, Minute Maid over the past week?’. One time/serving was defined as either a standard bottle or can (approximately 350 ml) or a regular cup (approximately 250 ml). Following the WHO guideline on the intake of free sugars for reduction in the risk of chronic diseases in children (29), SSB was categorized into two groups: 1) <1 time/day as low SSB; 2) ≥1 time/day as high SSB.

SST was assessed by asking the question ‘How long did you use electronic devices over the past week, including watching television, using computers, smart phone and tablet, and playing video games?’. The time spent on electronic devices meeting the SST guideline requires less than 2 hours daily according to the Canadian 24-Hour Movement Guidelines (24HGs) (9). SST was recorded as: 1) <2 hours/day as appropriate SST, 2) ≥2 hours/day as excessive SST.

All students reported their average SLD every night for the past week. SLD was categorized based on the recommendation proposed by the National Sleep Foundation: 1) very short SLD (< 7 and < 6 hours per night for students aged ≤13 years and students aged 14–19 years, respectively); 2) short SLD (7–8 and 6–7 hours per night for students aged ≤13 and students aged 14–19 years, respectively); 3) recommended SLD (9–11 and 8–10 hours per night for students aged ≤13 years and students aged 14–19 years, respectively); and 4) long SLD (> 11 and > 10 hours per night for students aged ≤13 years and students aged 14–17 years, respectively) (30). Due to small proportions of participants with very short (3.97%) and long SLD (0.81%), we combined very short with short SLD, and long with sufficient SLD, and classified SLD into two groups: 1) short SLD, 2) sufficient SLD.

### 2.3 Covariates

Body weight status was assessed based on body mass index (BMI) z-score. Body weight status in Chinese students was classified into four groups: underweight (UW), normal weight (NW), overweight (OW) and obesity (OB), using sex- and age- specific reference data from WHO (31).

Students were asked to report information on demographic [sex, age (<15 and ≥15), school type (junior middle school, senior middle school/vocational high school), residence area (urban, urban-rural junction/rural areas) and family structure (single-parent, both-parent, grandparents, parents and grandparents, and others)], the levels of moderate-to-vigorous physical activity (MVPA: 0-2, 3-6, 7 days/week) and junk food consumption for the past week was categorized as: never, <1 time/day, ≥1 time/day.

### 2.4 Statistical analysis

Because of the relatively low prevalence of depressive symptoms (2022: 14.81% and 2023: 17.52%) and specific lifestyle behavior combinations (e.g. excessive SST: 10.40% in 2022, 8.81% in 2023; low SSB: 7.53% in 2022, 9.89% in 2023) (**Supplementary Table 2**), statistical power to examine sex-specific associations in single survey year was limited. We, therefore, pooled two survey samples for data analysis. The samples were divided into sub-groups according to sex. The numbers, percentages or 95% confidence interval (CI), and means with standard deviation (SD) were presented for category variables and continuous variables respectively. The subgroup differences in the percentage and prevalence were tested using the Pearson Chi-square (χ²) test. Student’s t test was used to compare means between sexes.

To evaluate potential selection bias, we compared key characteristics between included and excluded participants. A sensitivity analysis was undertaken to validate CES-D used for Chinese adolescents. Cronbach’s alpha of greater than 0.85 identifies internal reliability of CES-D (28). In this study, Cronbach’s alpha of 0.91 indicated that the CES-D questionnaire has high reliability for adolescents.

Independent associations of SSB, SST and SLD with depressive symptoms were examined using multilevel logistic regression with a random intercept at the school level via two models: 1) crude model; 2) adjusted model: adjusting for demographic, PA levels, junk food consumption, health status (heart diseases, hypertension, diabetes and allergic diseases), BMI, and two other explanatory variables included in the model.

Multilevel logistic regression with a random intercept at the school level was used to examine the association between depressive symptoms and the combined lifestyle patterns of SSB, SST and SLD adjusting for demographic, PA levels, junk food consumption, health status and BMI. The healthy and unhealthy lifestyle patterns are defined as behaviors combinations that follow current public health recommendations for adolescent (9, 29, 30). The healthy lifestyle pattern is characterized by low SSB consumption, appropriate SST and sufficient SLD. Conversely, the unhealthy pattern is characterized by high SSB consumption, excessive SST and short SLD. The healthy pattern (Pattern 1) was the reference category in the model. Because of the research question, sex-stratified analysis was conducted to examine sex-specific associations between lifestyle patterns and depressive symptoms.

Results were considered statistically significant at a two-tailed level of 0.05. Statistical analysis was conducted using the STATA statistical software package version 18 (2021).

## 3 Results

### 3.1 Study population and characteristics

Comparison of the key characteristics between included and excluded participants revealed minor differences supporting the robustness of our results (**Supplementary Table 1**). The average age was 14.97 in the total adolescents (boys: 52.14%) with mean CES-D score of 9.38. Around 55.31% of adolescents were older adolescents (≥15 years), 46.81% were junior high school students, 69.93% lived in urban areas, 59.85% were from both-parent families, and 10.76% of adolescents met PA recommendation (**Table 1**). The differences in the percentages of demographic, MVPA levels and body weight status were significant between boys and girls. The mean values of weight, height and BMI were higher in boys, while the mean score of CES-D was higher in girls (P<0.001).

**Table 1:** General characteristics of adolescents.

Characteristics	Total	Boys	Girls	P*
	n (%)			
Sex				
Boys	9,937 (52.14)	–	–	
Girls	9,120 (47.86)	–	–	
Age groups				
<15	8,516 (44.69)	4,535 (45.64)	3,981 (43.65)	0.105
≥ 15	10,541 (55.31)	5,402 (54.36)	5,139 (56.35)	
School type				
Junior	8,920 (46.81)	4,761 (47.91)	4,159 (45.60)	0.001
Senior	10,137 (53.19)	5,176 (52.09)	4,961 (54.40)	
Family member				0.048
Single-parent	2,168 (11.38)	1,091(10.98)	1,077 (11.81)	
Both-parent	11,405 (59.85)	5,962 (60.00)	5,443 (59.68)	
Grandparents	1,175 (6.17)	588 (5.92)	587 (6.44)	
Parents and grandparents	4,127 (21.66)	2,209 (22.23)	1,918 (21.03)	
Others	182 (0.96)	87 (0.88)	95 (1.04)	
Area of residence				0.143
Urban	13,326 (69.93)	6,995 (70.39)	6,331 (68.42)	
Urban-rural junction/rural areas	5,731 (30.07)	2,942 (29.61)	2,789 (30.58)	
Moderate-to-vigorous physical activity (days/week)				<0.001
0-2	8,669 (45.49)	4,021 (40.46)	4,648 (50.96)	
3-6	8,338(43.75)	4,525 (45.54)	3,813 (41.81)	
7	2,050 (10.76)	1,391 (14.00)	659 (7.23)	
Body weight status				<0.001
Underweight	2,919 (15.32)	1,428 (14.37)	1,491 (16.35)	
Normal weight	1,1145 (58.48)	5,207 (52.40)	5,938 (65.11)	
Overweight	3,337 (17.51)	2,083 (20.96)	1,254 (13.75)	
Obesity	1,656 (8.69)	1,219 (12.27)	437 (4.79)	
Disease history				
Heart disease	11 (0.06)	7 (0.07)	4 (0.04)	0.445
Hypertension	10 (0.05)	6 (0.06)	4 (0.04)	0.619
Diabetes	2 (0.01)	2 (0.02)	0 (0.00)	0.175
Allergic diseases	81 (0.43)	31 (0.31)	50 (0.55)	0.012
Mean (SD)				
Age (Years)	14.97 (1.77)	14.95 (1.78)	14.99 (1.76)	0.145
Weight (kg)	58.18 (14.00)	62.38 (15.31)	53.61 (10.69)	<0.001
Height (cm)	165.24 (8.98)	169.43 (9.12)	160.67 (6.15)	<0.001
BMI (kg/m^2^)	21.12 (4.02)	21.52 (4.29)	20.67 (3.65)	<0.001
CES-D	9.38 (8.30)	9.07 (8.00)	9.72 (8.60)	<0.001

BMI, body mass index; CES-D, Centre for Epidemiological Studies-Depression; SD, standard deviation;.

*The difference in categories between boys and girls was examined by Pearson Chi-square tested and the difference in mean values was tested by Student's t test.

### 3.2 The prevalence of depressive symptoms

The overall prevalence of depressive symptoms was 16.29% (95% CI: 15.76%**–**16.81%), and significantly higher in girls than boys (14.54%, 95% CI: 13.79%**–**15.18% vs. 18.27%, 95% CI: 17.49%**–**19.07%; P <0.001) and in senior high schools adolescents than in junior high school students (13.98%, 95% CI: 13.27%**–**14.72% vs. 18.32%, 95% CI: 17.58%**–**19.09%; P <0.001). Regarding lifestyle behaviors, the prevalence of depressive symptoms was significantly higher among adolescents with low SSB consumption (28.32%, 95% CI: 26.21%**–**30.52%), excessive SST (27.25%, 95% CI: 25.28%**–**29.35%) and short SLD (18.50%, 95% CI: 17.82%**–**19.20%) (**Table 2**). Stratified by sex, boys consuming low SSB and spending excessive SST on electronic devices, reported the higher prevalence of depressive symptoms than girls (P<0.001). Compared to boys, the higher prevalence of depressive symptoms was found among girls with short SLD (P<0.001).

**Table 2:** The prevalence of depressive symptoms stratified by the level of lifestyle behaviors.

	Total*				Boys*					Girls*			
Lifestyle behaviors	Non-depressive symptoms	Depressive symptoms	Prevalence of depressive symptoms		Non-depressive symptoms	Depressive symptoms	Prevalence of depressive symptoms		Non-depressive symptoms		Depressive symptoms	Prevalence of depressive symptoms	
	n (%)		%	95% CI	n (%)		%	95% CI	n (%)			%	95% CI
Sugar-sweetened beverage													
Low	1205 (7.55)	476 (15.34)	28.32	26.21,30.52	788 (9.27)	269 (18.71)	25.45	22.89, 28.20	417 (5.59)		207 (12.42)	33.17	29.53, 36.98
High	14748 (92.45)	2628 (84.66)	15.12	14.60,15.66	7711 (90.73)	1169 (81.29)	13.16	12.47, 13.89	7037 (94.41)		1459 (87.58)	17.17	16.38, 18.00
Screen-based sedentary behavior													
Appropriate	14632 (91.72)	2609 (84.05)	15.13	14.61,15.68	7696 (90.55)	1177 (81.85)	13.27	12.58,13.99	6936 (93.05)		1432 (85.95)	17.11	16.32, 17.93
Excessive	1321 (8.28)	495 (15.95)	27.25	25.28, 29.35	803 (9.45)	261 (18.15)	24.53	22.05, 27.20	518 (6.95)		234 (14.05)	31.12	27.93,34.52
Sleep duration													
Short	9902 (62.07)	2248 (72.42)	18.50	17.82, 19.20	5053 (59.45)	991 (68.92)	16.40	15.49,17.35	4849 (65.05)		1257 (75.45)	20.59	19.58, 21.63
Sufficient	6051 (37.93)	856 (27.58)	12.39	11.64, 13.19	3446 (40.55)	447 (31.08)	11.48	10.51, 12.53	2605 (34.95)		409 (24.55)	13.57	12.39, 14.85

CI, confidence interval.

*The difference between non-depressive symptoms and depressive symptoms was examined by Pearson Chi-square test (χ²). P values <0.001 for total adolescents, boys and girls.

### 3.3 Independent associations of sugar-sweetened beverage consumption, screen-based sedentary time and sleep duration with depressive symptoms

SSB, SST and SLD were significantly associated with depressive symptoms in the crude model (**Table 3**). In adjusted model, excessive SST and short SLD were associated with higher odds of depressive symptoms in both boys (excessive SST- AOR: 2.10, 95% CI: 1.77–2.48; short SLD-AOR: 1.50, 95% CI: 1.29–1.75) and girls (excessive SST- AOR: 2.02, 95% CI: 1.62–2.53; short SLD-AOR: 1.66, 95% CI: 1.46–1.89). Conversely, high SSB consumption was associated with a lower risk of depressive symptoms in both boys (AOR: 0.57, 95% CI: 0.47–0.68) and girls (AOR: 0.52, 95% CI: 0.42–0.66).

**Table 3:** Sex-stratified independent associations of sugar-sweetened beverage consumption, screen-based sedentary time, and sleep duration with adolescent depressive symptoms: results from multilevel logistic regression with a school-level random intercept.

	Boys				Girls			
	Crude model		Adjusted model^a^		Crude model		Adjusted model^a^	
Lifestyle behaviors	OR	95% CI	AOR	95% CI	OR	95% CI	AOR	95% CI
Sugar-sweetened beverage								
Low	1		1		1		1	
High	0.44	0.37,0.53	0. 57	0.47,0.68	0.42	0.33,0.53	0.52	0.42,0.66
Screen-based sedentary behavior								
Appropriate	1		1		1		1	
Excessive	2.14	1.79,2.56	2.10	1.77,2.48	2.17	1.79,2.65	2.02	1.62,2.53
Sleep duration								
Sufficient	1		1		1		1	
Short	1.51	1.25,1.83	1.50	1.29,1.75	1.65	1.42,1.91	1.66	1.46,1.89

AOR, odds ratio; CI, confidence interval; OR, odds ratio.

^a^ Adjusting for demographic, PA levels, junk food consumption, health status and BMI and two other explanatory variables.

### 3.4 Association between combined lifestyle patterns and depressive symptoms

**Figure 2:**
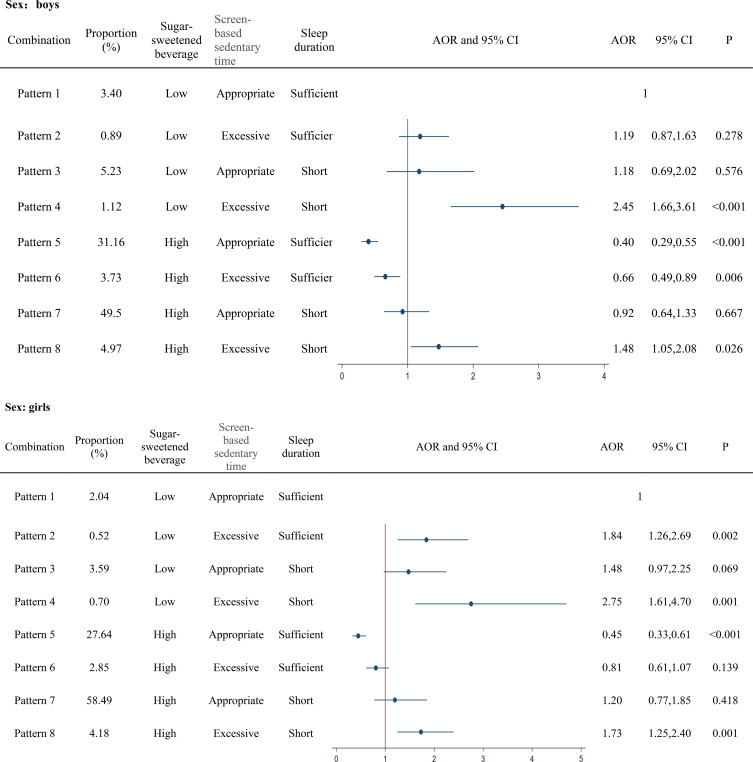
Sex- specific association between combinations of sugar-sweetened beverage consumption, screen-based sedentary time and sleep duration, and adolescent depressive symptoms. AOR, odds ratio; CI, confidence interval.

Pattern 4 was associated with higher odds of depressive symptoms (boy- AOR: 2.45, 95% CI: 1.66–3.61; girl- AOR: 2.75, 95% CI: 1.61–4.70), followed by Pattern 8 (boy- AOR:1.48, 95% CI: 1.05–2.08; girl-AOR: 1.73, 95% CI: 1.25–2.40) among both boys and girls (**Figure 2**). However, Pattern 5 was associated with a lower risk of depressive symptoms among both boys and girls (boy-AOR: 0.40, 95% CI: 0.29–0.55; girl- AOR: 0.45, 95% CI: 0.33–0.61). In female adolescents, Pattern 2 was associated with greater depressive symptoms (AOR: 1.84, 95% CI: 1.26–2.69), while Pattern 6 was negatively associated with depressive symptoms in male participants (AOR: 0.66, 95% CI: 0.49–0.89).

## 4 Discussion

This representative study is the first study to explore sex-specific associations of combined those lifestyle behaviors with depressive symptoms in Chinese adolescents. Girls reported a higher prevalence of depressive symptoms. Our findings indicate that associations of the combination of SSB consumption, SST, and SLD with depressive symptoms differed by sex.

The overall prevalence of depressive symptoms in our study was slightly higher than that reported in our previous study (15.27%), but slightly lower than the prevalence reported in a study conducted in Eastern China (17.8%) and much lower than adolescents at the national level in China (24.3%) (5, 15, 32). In line with our previous study, girls had a higher prevalence of depressive symptoms than male participants (15). Likewise, previous Chinese (17, 32, 33) and foreign studies (34–36) concluded a higher prevalence of depressive symptoms and other mental disorders in female adolescents. Several factors such as sex hormones, genetic factors, interpersonal relationships within family and academic pressure may explain the higher risk of depressive symptoms in girls (37–39). Notably, female adolescents in China experience greater academic pressure than their male counterparts. A national survey from China Youth & Children Research Center (2010) showed that girls spent significantly more time on homework and extracurricular study, with 47.4% of girls spending more than two hours daily on homework and 35.1% engaging in additional study beyond regular schoolwork (40). Spending more than two hours per day on homework has been identified as a risk factor for depressive symptoms among Chinese adolescents (41), indicating academic pressure may partially explain the increased risk in girls. Another study revealed that adolescent girls may experience stronger expectations of being a ‘good student’ and an ‘obedient daughter’, leading to greater internalized stress (42). Moreover, compared to Chinese boys, girls tend to ruminate more on negative emotions and academic setbacks (43), whereas boys may be more likely to engage in externalizing behaviors such as conduct problems (42, 44).

With rapid economic growth in China, adolescents’ lifestyle behaviors transitioned towards sedentary lifestyle with an increase in the use of electronic devices, a reduction in SLD and unhealthy dietary patterns/Western diets (8). Evidence shows that healthy lifestyle behaviors such as low SSB consumption, low SST and sufficient SLD are associated with lower odds of depressive symptoms in adolescents (11, 17, 21). In line with a previous study, our results indicated independent associations of low SSB consumption, excessive SST and short SLD with depressive symptoms (45).

To date, no study among Chinese adolescents has examined the joint association of SSB, SST, and SLD with depressive symptoms. However, recent evidence from China highlights that unhealthy lifestyle patterns are linked to mental health risks (23, 46). A recent cross-sectional study conducted in Shanghai primary and secondary schools reported that adolescents with any of three adverse behavioral combinations, including insufficient SLD, long SST, poor diet quality, and physical inactivity, had 4.7-fold higher odds of depression (46). Similarly, another cross-sectional study in Chinese adolescents in Ningbo indicated that the combined lifestyle pattern of low level of MVPA, excessive ST and short SLD was associated with 1.7 times higher odds of depressive symptoms (23). Although these studies did not specifically assess SSB frequency, they all indicate that co-occurring unhealthy lifestyle behaviors are associated with poorer mental health in Chinese adolescents.

Sex differences have been reported in mental health symptoms associated with lifestyle behaviors (47). Consistent with one recent study conducted on South Korean adolescents in 2021 (45), low to high SSB consumption combined with excessive SST and short SLD (Pattern 3 and Pattern 8) in our study were associated with a greater increase in depressive symptoms in both boys and girls. Importantly, Pattern 4 was associated with the highest odds of depressive symptoms, followed by Pattern 7. Another finding showed that Pattern 5 was associated with a lower risk of depressive symptoms in both boys and girls. Therefore, SSB consumption may be an important factor associated with depressive symptoms (48). Our results indicated that SSB consumption (≥1 time/day) was independently associated with low odds of depressive symptoms in both boys and girls. Mrug et al. reported a similar finding from a longitudinal study that SSB at age 13 predicted fewer depressive symptoms two years later (22). In contrast, other studies suggested that high SSB (16 times or more/week) consumption, or early adolescent (at ages 11) consumption were associated with an increased risk of greater depressive symptoms and aggressive behaviors (22, 49). The discrepancy may be explained by differences in dietary culture. Unlike Western dietary culture where carbonated soft drinks dominate SSB intake, the majority of Chinese adolescents consume tea-based beverages (e.g. milk tea) and fruit-flavored drinks, many of which contain bioactive compounds (e.g., tea polyphenols) with potential mood-modulating effects (50, 51). Another possibility is that adolescents already experiencing depressive symptoms may have reduced appetite or altered food preferences, leading to lower consumption of SSBs (52). Nevertheless, our results are from cross-sectional data, and the reasons for the inconsistent results remain unclear. Experimental studies suggested that acute sugar intake may transiently modulate dopamine and serotonin levels, thus, influence mood (53, 54). Self-reported habitual consumption in a cross-sectional survey cannot reliably capture short-term neurochemical effects. The inverse association in our study may be attributed to methodological limitations including report bias, measurement error, reverse causality or temporal ambiguity.

Noteworthily, our results revealed that excessive SST combined with co-existing low SSB consumption and sufficient SLD (Pattern 2) was associated with higher odds of depressive symptoms in girls. In contrast, excessive SST together with high SSB and sufficient SLD (Pattern 6) was observed to be associated with lower odds of depressive symptoms in boys. Screen-based sedentary behavior was a stronger factor associated with mental health in female adolescents. A recent systematic review indicated that girls are more susceptible to depressive symptoms because girls tend to have sedentary lifestyles, which may be attributed to a combination of physiological, psychological, and behavioral factors (55). During a dynamic and sensitive period, adolescents undergo dramatic changes due to sex hormones (e.g., estrogen in girls, testosterone in boys. Estrogen fluctuations in girls are linked to stronger emotional responses and increased susceptibility to mood disorders, potentially increasing vulnerability to specific patterns of social media use (56). While testosterone in boys may promote conduct behaviors and reduce stress reactivity, possibly lessening emotional distress, even among them with high SSB consumption and screen exposure. Girls tend to exhibit greater sensitivity to social evaluation and media influence, and are more prone to adopting internalizing coping styles, which can be negatively associated with youth mental health. Conversely, boys are more likely to employ problem-solving oriented styles that may distract them from screen use, which are associated with reduced negative emotional effects of screen use. These differences can be associated with distinct screen-use patterns, where girls tend to prefer socially oriented, image-focused platforms, and are more likely to spend >2 hours/day on social networking sites, whereas boys more often engage in intensive online or video gaming (55, 57).

Another main finding in our study was that sufficient SLD interacted with high SSB consumption and excessive SST (Pattern 6) was only associated with weaker depressive symptoms in boys. Therefore, SLD may be linked to a lower prevalence of depressive symptoms in adolescents, highlighting its possible role as an indicator of better mental well-being. In line with our previous study, insufficient SLD was independently associated with a higher risk of depressive symptoms in both sexes (15). A recent study recruiting 1,283 adolescents from secondary schools in the United Kingdom using the Mood and Feelings Questionnaire revealed that less SLD was cross-sectionally associated with higher levels of depressive symptoms (58). Insufficient SLD has been reported to be associated with altered secretion of growth hormones, which is most likely to be linked to higher stress levels in adolescents (59). Higher levels of accumulated stress would be associated with the deterioration of mental health and a greater likelihood of psychiatric symptoms (59). Compared to girls, boys generally engage in higher levels of MVPA, particularly through outdoor sports, would be concurrently associated with lower cortisol levels and better sleep outcomes, which in turn can be associated with better mental health and lower risk of mental disorders (60, 61).

Moreover, Pattern 6 (3.21%) and Pattern 2 (0.71%) were only observed significantly associated with depressive symptoms in boys and girls, respectively. An explanation for this may be low proportions of adolescents influenced the exact results of associations.

This representative school-based study with a large sample size was to investigate the association of combinations of SSB, SST, and SLD with depressive symptoms in Chinese adolescents. Our results highlight key lifestyle behaviors associated with better mental health in adolescents, providing a basis for future public health strategies. However, this study still has several limitations. First, causality cannot be inferred due to the nature of cross-sectional study design. Second, this study was conducted in Ningbo, a relatively developed coastal city in the Yangtze River Delta of China. Therefore, our finding may not be generalizable to adolescents in rural or less-developed regions in China, especially central and western China, where socioeconomic conditions, educational resources, and lifestyle environments differ substantially. Nevertheless, given the similarities in lifestyle behaviors, economic status, urbanization level and the prevalence of depressive symptoms across the region of the Yangtze River Delta, our results are more likely representative of adolescents in these areas. Third, CES-D score might be underestimated due to psychological biases although depressive symptoms were assessed through an internationally standardized questionnaire. Fourth, since self-reported data relied on memory, it might have introduced potential recall and social desirability biases, resulting in misestimation of frequency of SSB consumption, the duration of SST and SLD. Therefore, it may have affected the accuracy of the association between depressive symptoms and the combined patterns of SSB, SST and SLD. Fifth, the dichotomization of lifestyle behaviors may oversimplify complex exposure patterns and potentially mask real dose–response relationships. In our study, SSB, SST and SLD were categorized according to standard public health thresholds (≥1 time/day for SSB based on WHO, ≥2 hours/day for screen time based on 24HGs, and age-specific thresholds for SLD recommended by the National Sleep Foundation) (9, 29, 30). Although these valid and standardized categorizations can enhance comparability with national and international adolescent health surveys and promote public health messaging, such binary classifications inevitably reduce complex behavioral continua. To assess the robustness of our findings, we conducted a sensitivity analysis using alternative thresholds. The results showed that the associations remained statistically significant with minor changes in the estimates and confidence intervals that (**Supplementary Table 5**). Therefore, this indicates that our findings using the cut-off points defined by public health thresholds are robust. Another limitation is that, although pooling 2022 and 2023 data increased power for examining sex-specific associations of combined lifestyle patterns with depressive symptoms, it may mask slight temporal variations in these lifestyle behavior-mental health relationships. However, year-stratified sensitivity analysis revealed similar distributions of lifestyle behaviors and robust associations across both survey waves (**Supplementary Tables 2–4**), supporting the robustness of our main findings derived from pooled analysis. Moreover, although eight lifestyle patterns were defined based on public health thresholds, some combined patterns had small sample sizes, which may reduce the precision of estimated associations for those subgroups. A sensitivity analysis using alternative thresholds was conducted to re-categorize the combined pattern for evaluating the robustness of our findings (**Supplementary Table 6**). The comparison indicated that the associations of the combined patterns derived from public health guidelines were mostly robust. We acknowledge that our cross-classification approach is that it assumes equal contribution of each behavior to the overall lifestyle patterns, which may oversimplify their relative association with mental health. Future studies should employ more comprehensive analytical approaches such as weighted overall scores, latent class analysis or machine learning approaches to better capture real-word adolescent behaviors and to identify novel lifestyle patterns associated with the risk of mental health. Finally, the specific types of SSB (e.g. carbonated soft drinks, sweetened tea, fruit-flavored beverages) and contents of screen-based activities (e.g., gaming, social media, online learning) were not measured in this study. Additional unavailable factors including family-related socioeconomic factors (e.g., parental education, household income, academic pressure, and social support), psychological factors (e.g., academic stress and peer relationships) and social and cultural environments were not collected in this National Surveillance Program on Common Morbidities and Health-Related Risk Factors among Students. These unmeasured variables, which are known to be associated with adolescent mental health, should be collected in future studies. Consequently, residual confounding cannot be ruled out and might have influenced the accuracy of associations. For future studies, well-designed longitudinal studies, with repeated measures of both lifestyle behaviors and mental health outcomes are needed to clarify associations, identify critical risks of behaviors, and assess whether modifying specific lifestyle factors may reduce the incidence of depressive symptoms among Chinese adolescents.

## 5 Conclusion

Our results showed sex differences in the prevalence of depressive symptoms and lifestyle behaviors. Depressive symptoms were more prevalent in girls. SSB consumption, SST and SLD were independently associated with depressive symptoms in both sexes. In addition, the combinations of SSB consumption, SST and SLD associated with depressive symptoms showed sex-specific patterns. The findings suggest that the potential value of sex-specific public health strategies in future family- and school-based health programs. Specifically, these sex-specific strategies could include reducing screen time (particularly social media use) for both sexes, with additional emphasis on outdoor physical activity and consistent sleep schedules for girls, and on ensuring sufficient sleep duration for boys. All these strategies are aimed at promoting healthy lifestyle behaviors and preventing mental disorders in Chinese adolescents.

## Data Availability

The raw data supporting the conclusions of this article are not publicly available due to privacy and ethical restrictions. The data will be made available upon reasonable request to the corresponding authors.
