# Supplementary material for: Sex- specific interplay of combined lifestyle patterns and their association with depressive symptoms among Chinese adolescents: a school-based cross-sectional study
Source: Front Psychiatry. 2026 May 12;17:1747059. doi: 10.3389/fpsyt.2026.1747059 (PMC13201451; doi:10.3389/fpsyt.2026.1747059)
Supplement: Supplementary file 1 [file Table1.docx]

| **Supplementary Table 1.** Sensitivity analyses: Comparison of general characteristics between included and excluded participants | | | |
| --- | --- | --- | --- |
| Characteristics | Excluded participants (n=4,342) | Included participants (n=19,057) | P |
|  | n (%) | |  |
| Sex |  |  | 0.375 |
| Boys | 2,278 (52.46) | 9,937 (52.14) |  |
| Girls | 2,064 (47.54) | 9,120 (47.86) |  |
| Age groups |  |  | 0.191 |
| <15 | 1,857 (42.77) | 8,516 (44.69) |  |
| ≥ 15 | 2,485 (57.23) | 10,541 (55.31) |  |
| Area of residence |  |  | <0.001 |
| Urban | 3,414 (78.63) | 13,326 (69.93) |  |
| Urban-rural junction/rural areas | 928 (21.37) | 5,731 (30.07) |  |
|  | Mean (SD) | |  |
| Age (Years) | 15.33 (2.12) | 14.97 (1.77) | <0.001 |
| Weight (kg) | 58.63 (14.27) | 58.18 (14.00) | 0.062 |
| Height (cm) | 165.73 (9.14) | 165.24 (8.98) | 0.001 |
| BMI (kg/m^2^) | 21.15 (4.02) | 21.12 (4.02) | 0.670 |
| *The difference in categories between excluded and included participants was examined by Pearson Chi-square tested and the difference in mean values was tested by Student's t test. | | | |
