# Supplementary material for: Sex- specific interplay of combined lifestyle patterns and their association with depressive symptoms among Chinese adolescents: a school-based cross-sectional study
Source: Front Psychiatry. 2026 May 12;17:1747059. doi: 10.3389/fpsyt.2026.1747059 (PMC13201451; doi:10.3389/fpsyt.2026.1747059)
Supplement: Supplementary file 2 [file Table2.docx]

| **Supplementary Table 2.** Lifestyle behaviors and depressive symptoms in adolescents by survey year | | | |
| --- | --- | --- | --- |
| Items | 2022 (n=8,645) | 2023 (n=10,412) | P |
|  | n (%) | |  |
| Depressive symptoms |  |  | <0.001 |
| No | 7,365 （85.19） | 8,588 (82.48) |  |
| Yes | 1,280 (14.81) | 1,824 (17.52) |  |
| Sugar-sweetened beverage |  |  | <0.001 |
| Low | 651 7.53 | 1,030 9.89 |  |
| High | 7,994 92.47 | 9,382 90.11 |  |
| Screen-based sedentary time |  |  | <0.001 |
| Appropriate | 7,746 89.60 | 9,495 91.19 |  |
| Excessive | 899 10.40 | 917 8.81 |  |
| Sleep duration |  |  | <0.001 |
| Short | 5,290 61.19 | 6,860 65.89 |  |
| Sufficient | 3,355 38.81 | 3,552 34.11 |  |
| *The difference between non-depressive symptoms and depressive symptoms was examined by Pearson Chi-square test (Χ²). | | | |
