# Supplementary material for: Sex- specific interplay of combined lifestyle patterns and their association with depressive symptoms among Chinese adolescents: a school-based cross-sectional study
Source: Front Psychiatry. 2026 May 12;17:1747059. doi: 10.3389/fpsyt.2026.1747059 (PMC13201451; doi:10.3389/fpsyt.2026.1747059)
Supplement: Supplementary file 3 [file Table3.docx]

| **Supplementary Table 3.** Sensitivity analyses: Sex-stratified independent associations of sugar-sweetened beverage consumption, screen-based sedentary time, and sleep duration with adolescent depressive symptoms across survey years^a^ | | | | | | | | | |
| --- | --- | --- | --- | --- | --- | --- | --- | --- | --- |
| Lifestyle behaviors | 2022 (n=8,645) | | | |  | 2023 (10,412) | | | |
|  | Boys | | Girls | |  | Boys | | Girls | |
|  | AOR | 95% CI | AOR | 95% CI |  | AOR | 95% CI | AOR | 95% CI |
| Sugar-sweetened beverage |  |  |  |  |  |  |  |  |  |
| Low | 1 | | | |  | 1 | | | |
| High | 0.49 | 0.36,0.67 | 0.46 | 0.33,0.65 |  | 0.63 | 0.50,0.78 | 0.57 | 0.44,0.74 |
| Screen-based sedentary behavior |  |  |  |  |  |  |  |  |  |
| Appropriate | 1 | | | |  | 1 | | | |
| Excessive | 1.94 | 1.54,2.45 | 2.39 | 1.82,3.14 |  | 2.24 | 1.77,2.85 | 1.78 | 1.39,2.29 |
| Sleep duration |  |  |  |  |  |  |  |  |  |
| Sufficient | 1 | | | |  | 1 | | | |
| Short | 1.30 | 1.03,1.64 | 1.44 | 1.17,1.76 |  | 1.70 | 1.47,1.96 | 1.85 | 1.55,2.21 |
| AOR: odds ratio; CI: confidence interval | | | | | | | | | |
| ^a^ Multilevel logistic regression with a school-level random intercept was used to examine associations after adjusting for demographic, junk food consumption, PA levels, health status and BMI and two other explanatory variables | | | | | | | | | |
