# Supplementary material for: Sex- specific interplay of combined lifestyle patterns and their association with depressive symptoms among Chinese adolescents: a school-based cross-sectional study
Source: Front Psychiatry. 2026 May 12;17:1747059. doi: 10.3389/fpsyt.2026.1747059 (PMC13201451; doi:10.3389/fpsyt.2026.1747059)
Supplement: Supplementary file 4 [file Table4.docx]

| **Supplementary Table 4.** Sensitivity analyses: Sex-stratified independent associations of sugar-sweetened beverage consumption, screen-based sedentary time, and sleep duration with adolescent depressive symptoms across survey years^a^ | | | | | | | | | | | | |
| --- | --- | --- | --- | --- | --- | --- | --- | --- | --- | --- | --- | --- |
| Combination | Lifestyle behaviors | | | 2022 (n=8,645) | | | |  | 2023 (n=10,412) | | | |
|  |  |  |  | Boys | | Girls | |  | Boys | | Girls | |
|  | Sugar-sweetened beverage | Screen time (hour/d) | Sleep duration | AOR | 95% CI | AOR | 95% CI |  | AOR | 95% CI | AOR | 95% CI |
| Pattern 1 | Low | Appropriate | Sufficient | 1 |  | 1 |  |  | 1 |  | 1 |  |
| Pattern 2 | Low | Excessive | Sufficient | 0.87 | 0.49,1.55 | 1.88 | 1.00,3.56 |  | 1.49 | 1.01,2.20 | 1.84 | 1.07,3.17 |
| Pattern 3 | Low | Appropriate | Short | 0.89 | 0.45,1.74 | 1.79 | 0.95,3.37 |  | 1.44 | 0.69,3.01 | 1.09 | 0.56,2.14 |
| Pattern 4 | Low | Excessive | Short | 1.44 | 0.79,2.60 | 2.91 | 1.38,6.10 |  | 3.63 | 1.99,6.61 | 2.61 | 1.21,5.61 |
| Pattern 5 | High | Appropriate | Sufficient | 0.29 | 0.16,0.53 | 0.44 | 0.25,0.76 |  | 0.49 | 0.34,0.71 | 0.44 | 0.30,0.65 |
| Pattern 6 | High | Excessive | Sufficient | 0.45 | 0.27,0.76 | 0.71 | 0.43,1.18 |  | 0.86 | 0.61,1.22 | 0.89 | 0.61,1.29 |
| Pattern 7 | High | Appropriate | Short | 0.72 | 0.44,1.19 | 1.22 | 0.67,2.22 |  | 1.01 | 0.57,1.77 | 1.08 | 0.55,2.13 |
| Pattern 8 | High | Excessive | Short | 0.85 | 0.46,1.55 | 1.75 | 1.04,2.94 |  | 2.16 | 1.43,3.26 | 1.67 | 1.12,2.49 |
| AOR: odds ratio; CI: confidence interval | | | | | | | | | | | | |
| ^a^Multilevel logistic regression with a school-level random intercept was used to examine associations adjusting for demographic, junk food consumption, PA levels, health status and BMI explanatory variables | | | | | | | | | | | | |
