# Supplementary material for: Sex- specific interplay of combined lifestyle patterns and their association with depressive symptoms among Chinese adolescents: a school-based cross-sectional study
Source: Front Psychiatry. 2026 May 12;17:1747059. doi: 10.3389/fpsyt.2026.1747059 (PMC13201451; doi:10.3389/fpsyt.2026.1747059)
Supplement: Supplementary file 5 [file Table5.docx]

| **Supplementary Table 5.** Sex-stratified associations of lifestyle behaviors with depressive symptoms in adolescents: sensitivity analysis using alternative cut-off points | | | | |
| --- | --- | --- | --- | --- |
| Categorization 1 | Boys ^a^ | | Girls ^a^ | |
|  | AOR | 95% CI | AOR | 95% CI |
| Sugar-sweetened beverage (time/day) |  |  |  |  |
| No or <1 | 1 | | 1 | |
| ≥1 | 0. 57 | 0.47,0.68 | 0.52 | 0.42,0.66 |
| Screen-based sedentary behavior (hours/day) |  |  |  |  |
| <2 | 1 | | 1 | |
| ≥2 | 2.10 | 1.77,2.48 | 2.02 | 1.62,2.53 |
| Sleep duration |  |  |  |  |
| Short | 1 | | 1 | |
| Sufficient | 1.50 | 1.29,1.75 | 1.66 | 1.46,1.89 |
| Categorization 2 | AOR | 95% CI | AOR | 95% CI |
| Sugar-sweetened beverage (time/day) |  |  |  |  |
| No | 1 | | 1 | |
| >1 | 0.74 | 0.60,0.91 | 0.67 | 0.57,0.79 |
| Screen-based sedentary behavior (hours/day) |  |  |  |  |
| <1 | 1 | | 1 | |
| ≥1 | 1.53 | 1.33,1.76 | 1.60 | 1.35,1.89 |
| Sleep duration (hours/day) |  |  |  |  |
| <7 | 1 | | 1 | |
| ≥7 | 2.03 | 1.75,2.35 | 1.72 | 1.44,2.06 |
| AOR: odds ratio; CI: confidence interval | | | | |
| ^a^Multilevel logistic regression with a school-level random intercept was used to examine associations adjusting for demographic, junk food consumption, PA levels, health status and BMI and other two explanatory variables. | | | | |
| ^b^Sleep duration in categorization 1 was classified based on the recommendations by the National Sleep Foundation (30). | | | | |
