# Supplementary material for: Sex- specific interplay of combined lifestyle patterns and their association with depressive symptoms among Chinese adolescents: a school-based cross-sectional study
Source: Front Psychiatry. 2026 May 12;17:1747059. doi: 10.3389/fpsyt.2026.1747059 (PMC13201451; doi:10.3389/fpsyt.2026.1747059)
Supplement: Supplementary file 6 [file Table6.docx]

| **Supplementary Table 6.** Sensitivity Analysis: Comparison of associations between combined lifestyle patterns and adolescent depression using public health–based vs. percentile–based cut–off thresholds ^a^ | | | | | | | | | |
| --- | --- | --- | --- | --- | --- | --- | --- | --- | --- |
| Combination 1 | Lifestyle behaviors | | | Boys | | | Girls | | |
|  | SSB (time/day) | SST (hours/d) | Sleep duration^b^ | AOR | 95% CI | P | AOR | 95% CI | P |
| Pattern 1 | <1 | <2 | Sufficient | 1 | |  | 1 | |  |
| Pattern 2 | <1 | ≥2 | Sufficient | 1.18 | 0.85,1.65 | 0.321 | 1.84 | 1.22,2.77 | 0.004 |
| Pattern 3 | <1 | <2 | Short | 1.17 | 0.67,2.05 | 0.577 | 1.46 | 0.72,2.96 | 0.291 |
| Pattern 4 | <1 | ≥2 | Short | 2.47 | 1.55,3.92 | <0.001 | 2.71 | 1.47,5.00 | 0.001 |
| Pattern 5 | ≥1 | <2 | Sufficient | 0.40 | 0.30,0.54 | <0.001 | 0.45 | 0.32,0.65 | <0.001 |
| Pattern 6 | ≥1 | ≥2 | Sufficient | 0.65 | 0.49,0.86 | 0.002 | 0.81 | 0.57,1.15 | 0.241 |
| Pattern 7 | ≥1 | <2 | Short | 0.91 | 0.63,1.32 | 0.626 | 1.20 | 0.77,1.87 | 0.415 |
| Pattern 8 | ≥1 | ≥2 | Short | 1.49 | 1.06,2.08 | 0.020 | 1.72 | 1.15,2.59 | 0.008 |
| Combination 2 | SSB (time/day) | SST (hours/day) | Sleep duration  (hours/day) | AOR | 95% CI | P | AOR | 95% CI | P |
| Pattern 1 | No | <1 | >7 | 1 | |  | 1 | |  |
| Pattern 2 | No | ≥1 | >7 | 1.62 | 1.36,1.94 | <0.001 | 1.89 | 1.61,2.20 | <0.001 |
| Pattern 3 | No | <1 | ≤7 | 1.53 | 1.29,1.81 | <0.001 | 1.61 | 1.27,2.05 | <0.001 |
| Pattern 4 | No | ≥1 | ≤7 | 2.47 | 1.98,3.09 | <0.001 | 3.10 | 2.41,3.99 | <0.001 |
| Pattern 5 | >1 | <1 | >7 | 0.48 | 0.34,0.70 | <0.001 | 0.49 | 0.37,0.66 | <0.001 |
| Pattern 6 | >1 | ≥1 | >7 | 1.18 | 0.91,1.52 | 0.207 | 1.26 | 0.95,1.68 | 0.104 |
| Pattern 7 | >1 | <1 | ≤7 | 1.07 | 0.72,1.61 | 0.734 | 1.31 | 0.98,1.77 | 0.069 |
| Pattern 8 | >1 | ≥1 | ≤7 | 2.48 | 1.62,3.78 | <0.001 | 2.77 | 2.00,3.82 | <0.001 |
| AOR: odds ratio; CI: confidence interval; SSB: sugar-sweetened beverage; SST: screen-based sedentary time | | | | | | | | | |
| ^a^Multilevel logistic regression with a school-level random intercept was used to examine associations adjusting for demographic, PA levels, junk food consumption, health status and BMI explanatory variables | | | | | | | | | |
| ^b^Sleep duration in Combination 1 was classified based on the recommendations by the National Sleep Foundation (30). | | | | | | | | | |
